# Caveolin-2 associates with intracellular chlamydial inclusions independently of caveolin-1

**DOI:** 10.1186/1471-2334-4-23

**Published:** 2004-07-22

**Authors:** Wilmore C Webley, Leonard C Norkin, Elizabeth S Stuart

**Affiliations:** 1 Department of Microbiology, University of Massachusetts – Amherst, MA 01003, USA

## Abstract

**Background:**

Lipid raft domains form in plasma membranes of eukaryotic cells by the tight packing of glycosphingolipids and cholesterol. Caveolae are invaginated structures that form in lipid raft domains when the protein caveolin-1 is expressed. The *Chlamydiaceae *are obligate intracellular bacterial pathogens that replicate entirely within inclusions that develop from the phagocytic vacuoles in which they enter. We recently found that host cell caveolin-1 is associated with the intracellular vacuoles and inclusions of some chlamydial strains and species, and that entry of those strains depends on intact lipid raft domains. Caveolin-2 is another member of the caveolin family of proteins that is present in caveolae, but of unknown function.

**Methods:**

We utilized a caveolin-1 negative/caveolin-2 positive FRT cell line and laser confocal immunofluorescence techniques to visualize the colocalization of caveolin-2 with the chlamydial inclusions.

**Results:**

We show here that in infected HeLa cells, caveolin-2, as well as caveolin-1, colocalizes with inclusions of *C. pneumoniae *(Cp), *C. caviae *(GPIC), and *C. trachomatis *serovars E, F and K. In addition, caveolin-2 also associates with *C. trachomatis *serovars A, B and C, although caveolin-1 did not colocalize with these organisms. Moreover, caveolin-2 appears to be specifically, or indirectly, associated with the pathogens at the inclusion membranes. Using caveolin-1 deficient FRT cells, we show that although caveolin-2 normally is not transported out of the Golgi in the absence of caveolin-1, it nevertheless colocalizes with chlamydial inclusions in these cells. However, our results also show that caveolin-2 did not colocalize with UV-irradiated *Chlamydia *in FRT cells, suggesting that in these caveolin-1 negative cells, pathogen viability and very likely pathogen gene expression are necessary for the acquisition of caveolin-2 from the Golgi.

**Conclusion:**

Caveolin-2 associates with the chlamydial inclusion independently of caveolin-1. The function of caveolin-2, either in the uninfected cell or in the chlamydial developmental cycle, remains to be elucidated. Nevertheless, this second caveolin protein can now be added to the small number of host proteins that are associated with the inclusions of this obligate intracellular pathogen.

## Background

The *Chlamydiaceae *are gram-negative obligate intracellular bacterial pathogens that replicate entirely within membrane bound inclusions that develop from the phagocytic vacuoles in which they enter. By mechanisms not understood in detail, inclusions avoid fusing with endosomes or lysosomes that might destroy the pathogen [1-3]. Instead, the inclusions provide an intracellular niche in which the pathogen can survive and complete its developmental cycle. The initial pathogen-containing endocytic vacuoles co-associate with microtubules, and dynein, and traffic to a perinuclear region, ultimately residing at the microtubule organizing center and in close proximity to the Golgi [4,5]. Here, they intercept exocytic vesicles of the biosynthetic pathway that are derived from the Golgi and continue their developmental cycle [6-9].

There are two genera in the family of *Chlamydiaceae *[10]. The genus *Chlamydia *is comprised of the *C. trachomatis *species, which contains human pathogens, as well as the mouse and swine strains, *C. muridarum *and *C. suis *respectively, while the *Chlamydophila *genus is more diverse, consisting of six species. Of these, *C. pneumoniae*, and *C. psittaci *are relevant in human diseases. *C. trachomatis *is primarily a pathogen of humans, containing the trachoma biovariant (biovar), and the lymphogranuloma biovar (LGV). The mouse pneumonitis agent (MoPn), frequently used in vaccine work [11,12], is now classified separately as *C. muridarumn *[10]. Some serological variants (serovars) of the trachoma biovar primarily infect the urogenital tract (e.g., E, K, and F) and are the major cause of significant venereal disease in the United States [13]. Other, trachoma serovars primarily infect the conjunctiva (e.g., A, B, and C), causing trachoma, the major form of preventable blindness, worldwide [14]. The LGV biovar also causes chronic venereal disease, which differs clinically from the sexually transmitted disease caused by strains of the trachoma biovar. *C. pneumoniae*, another human pathogen, is now implicated in atherosclerosis and cardiac disease, as well as respiratory infection [15,16]. *C. psittaci *primarily infects birds, but can also cause disease in humans.

The mechanism by which chlamydiae enter host cells is not well defined, largely because of conflicting reports that these organisms can enter by both clathrin-dependent, as well as clathrin-independent pathways. Recently, we examined the entry mechanisms of representative members of each of the above *Chlamydia *species and biovars. Our concern was whether their entry might be mediated by the cholesterol and sphingolipid enriched micro-domains in host cell plasma membranes known as lipid rafts [17]. Application of biochemical criteria and confocal microscopy demonstrated that five of the ten strains we examined indeed enter by a mechanism that is dependent on intact lipid rafts [18,19]. These strains included *C. trachomatis *serovars E, F, and K, *C. pneumoniae *(strain AR39), and *C. caviae *(Guinea Pig Inclusion Conjunctivitis strain; GPIC). In contrast *, C. trachomatis *serovars A, B, C, biovar LGV (L2 strain), and MoPn do not enter via lipid rafts.

Caveolae are now considered to be a specialized kind of lipid raft. These 50–100 nm flask-shaped invaginations form in lipid rafts when the caveolae marker protein, caveolin-1, is expressed [reviewed in [20]]. In contrast, lipid rafts are ubiquitous entities that form regardless of caveolin-1 expression. The inclusion membranes of all of the chlamydial strains that entered via lipid rafts, as indicated by biochemical criteria, also appeared to acquire caveolin-1 at entry, and accumulated more of the protein during development in caveolin-1 expressing HeLa cells [18,19]. In contrast to the strains that entered by a raft-dependent process, none of the other strains examined acquired caveolin-1 at entry.

The fact that entry of some chlamydial strains is dependent on lipid rafts in general, rather than on the more specialized caveolae, was demonstrated using Fischer Rat Thyroid (FRT) cells, which do not express caveolin-1 or form caveolae. All ten strains that we examined were able to enter the caveolin-1 negative FRT cells, but not if those cells were treated with pharmacological agents that specifically disrupt lipid rafts [19].

The Golgi is a likely source of most caveolin-1 on the inclusions of those strains entering via lipid rafts. This is suggested by the fact that caveolin-1 immunostaining of those inclusions actually increases as they enlarge and mature, and is consistent with reports that caveolin-1 first becomes associated with raft domains in the Golgi, and subsequently is transported to the plasma membrane on vesicles derived from the Golgi [21-23]. Moreover, chlamydial inclusions are known to intercept vesicles of the biosynthetic pathway that originate from the Golgi, from which they acquire sphingolipids and perhaps other nutrients as well[6,7]. However, since inclusions of all strains appear to acquire sphingolipids from the Golgi, whereas only a subset of strains acquires caveolin-1, there may be more than one sorting process and class of vesicles with which *Chlamydia *can interact.

The caveolin gene family also contains caveolin-2 [24] which has 38% sequence identity with caveolin-1 and 58% similarity. Caveolin-1 and -2 are co-expressed in most cells, in which they form hetero-oligomeric complexes. These complexes form in the endoplasmic reticulum (ER), becoming associated with lipid rafts in the Golgi, from which they are transported to the plasma membrane. All of the putative functions of caveolae are thus far associated with caveolin-1, and the function of caveolin-2 is essentially unknown. Importantly, unlike caveolin-1, caveolin-2 can not induce caveolae to form in lipid rafts. An experimental finding that is particularly relevant to the current study is that caveolin-2 can not exit the Golgi in caveolin-1 negative FRT cells. However, it can exit when recombinant caveolin-1 is expressed [25,26]. Thus, formation of hetero-oligomeric complexes of caveolin-1 and -2 facilitates transport of caveolin-2 out of the Golgi and its sorting to the plasma membrane.

In the present study, we ask whether caveolin-2, like caveolin-1, might be associated with inclusions formed by particular chlamydial strains. Unexpectedly, we found that in HeLa cells, caveolin-2 was associated with inclusions of strains that did not enter via lipid rafts and that did not acquire caveolin-1 either at entry or at later times. These strains included *C. trachomatis *serovars A, B, and C. In the same cells, inclusions of *C. trachomatis *serovars E, F, and K, *C. pneumoniae *(A39), and *C. caviae *(GPIC) were marked by both of the caveolin proteins. Each of these eight strains acquired caveolin-2 even in caveolin-1 negative FRT cells. In addition, the ability of each of these strains to acquire caveolin-2 in the FRT cells was dependent on pathogen gene expression. Thus, chlamydiae appear to activate or induce an additional pathway for the transport of caveolin-2 that is independent of caveolin-1. Inclusions of two strains, LGV (L2) and MoPn, were not marked by either caveolin-1 or -2.

## Methods

### Chlamydial strains

*C. pneumoniae *AR39 (Cpn), *C. caviae, *Guinea Pig Inclusion Conjunctivitis (GPIC strain), *C. trachomatis *serovars A/Har-13, Har-36B, C/TW-3, E/VW-KX, F, K/VR887, mouse pneumonitis agent, (MoPn) and Lymphogranuloma venereum, (LGV 434) were grown in HeLa 229 cells without centrifuge assistance. Infectious elementary bodies (EBs) were purified by renografin (Squibb diagnostics, New Bronswick, NJ) density gradient centrifugation; alternatively lysates from infected cells were used to infect monolayers.

### Cell lines used

HeLa 229 cells were obtained from the American Type Culture collection. Fischer rat thyroid (FRT) cells were a gift from Dr. Michael P. Lisanti (Albert Einstein College of Medicine, Bronx, NY). Cells were grown in minimum essential medium with insulin (IMEMZO, Irvine Scientific, Santa Ana, CA) with 5% fetal bovine serum (FBS, Atlanta Biologicals, Norcross, GA.).

### Immunostaining of *Chlamydia *infected cells

HeLa 229 or FRT cells were grown to confluence on 12 mm coverslips in 24 well plates (Becton Dickinson Labware, Franklin Lakes, NJ). The cells were infected using the various chlamydial strains listed above at an MOI of 3.0 in complete cycloheximide overlay media (Bio-Whittaker, Walkersville, Md.) containing 10% FBS, 1X L-glutamine (CCOM) for 48 h at 37°C and 5% CO2. Coverslips with the cell monolayers were harvested, rinsed with phosphate buffered saline (PBS), fixed with 70% cold methanol, stored and subsequently immunostained following protocols similar to that described in detail previously [18,19]. Briefly, infected cells were immunostained with a guinea pig anti-chlamydia polyclonal antibody (Biomedia, Foster City, CA) and the monoclonal mouse anti-caveolin-2 antibody (Transduction Laboratories, Lexington, KY) for 1 h at 37°C. Following 4 washes with PBS, the bound antibodies were detected using a 1:50 dilution of TRITC-conjugated goat anti-guinea pig and FITC-conjugated goat anti-mouse secondary antibodies (Jackson Immuno Research, West Grove, PA). Following incubation for 1 h at RT and 4 rinses with (PBS), coverslips were mounted onto slides using Fluoromount-G (Southern Biotechnology Associates Inc., Birmingham, AL). Slides were examined at 600X using a Bio-Rad MRC-600 Laser Confocal Microscope system. Images were captured and as relevant, merged using the Confocal Assistant™ version 4.02 Image Processing Software.

### UV-treatment of EBs and infection of HeLa and FRT cells

*C. trachomatis *serovars A, B, C, E, F, LGV, *C. muridarum *(MoPn), *C. caviae *(GPIC), and *C. pneumoniae *EB concentrates were exposed to short wavelength ultraviolet light for 1 h in a Biosafety hood. A portion of the EBs used was removed before UV treatment to serve as a control for initial viability. The EBs were then diluted in CCOM to an MOI of 4.0 per cell and used to infect HeLa 229 and FRT cells grown to confluence on 12 mm coverslips in 24 well plates. Plates were incubated for 2 h at 37°C and 5% CO2. After the 2 h incubation, the inoculum was removed and replaced with fresh CCOM; cells were then re-incubated for 34 h. At the end of the incubation period, the coverslips with cells were rinsed with sterile PBS and fixed with methanol for 10 minutes as detailed above. Following fixation, the cells were immunostained with a polyclonal guinea pig anti-chlamydia and mouse monoclonal anti-caveolin-2 antibodies as described above. FITC-conjugated goat anti-mouse and TRITC-conjugated goat anti-guinea pig secondary antibodies were used respectively to visualize the caveolin-2 and EBs within the cells. Slides were examined at 600X with a Laser Confocal Microscope system and images processed as described above.

### Western blot of purified EBs and anti-Caveolin-2

*C. trachomatis *serovar K and *C. pneumoniae *AR39 purified EBs were electrophoresed on a 4–12% NuPAGE gel (Invitrogen Life technologies, Carlsbad, CA) using MES running buffer. The separated proteins were transferred to a PVDF membrane at 25 V for 1 h using a 1X Novex^® ^Tris Glycine buffer with 20% methanol in the XCell II™ Blot Module and a XCell *Surelock*™ Mini-Cell apparatus (Invitrogen). The blot was then rinsed with 0.1% BSA/PBS, blocked with 5% non-fat dry milk for 3 h, then rinsed and cut into two. One half was stained with a 1:200 dilution of mouse monoclonal anti-caveolin-2 antibody (Transduction Labs, Lexington, KY), while the second half was stained with a 1:100 dilution of a rabbit anti-*Chlamydia *for 2 h with shaking. The blot was then rinsed and a 1:1000 dilution of AP-conjugated goat anti-mouse secondary antibody added and incubated for 1 h at RT. Following several washes, a BCIP/NBT alkaline phosphatase substrate was added and the color allowed to develop.

## Results

### Caveolin-2 on chlamydial inclusions in caveolin-1 positive HeLa cells

**Figure 1:**
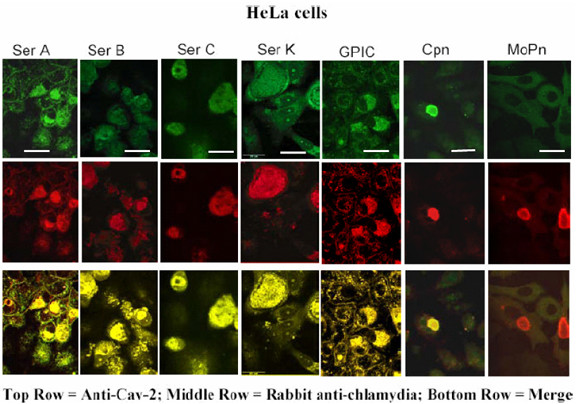
**Localization of chlamydial inclusions and caveolin-2 in HeLa cells**. HeLa 229 cells were infected with chlamydial strains for 48 h. Cells were fixed and double stained with a rabbit anti-*Chlamydia *and a mouse anti-caveolin-2 antibody. Inclusions of *C. pneumoniae *(AR39) Cpn, *C. Psittaci *(guinea pig inclusion conjunctivitis, GPIC strain), *C. trachomatis *serovars A/Har-13, Har36B, C/TW-3, K, (E/VW-KX and F not shown), are seen to co-localize with caveolin-2. Inclusions of *C. trachomatis *Mouse pnuemonitis agent (MoPn) and Lymphogranuloma venereum biovar (LGV 434) [not shown] do not colocalize with caveolin-2. Scale bar represents 25 μm and original magnification: 600X

**Figure 2:**
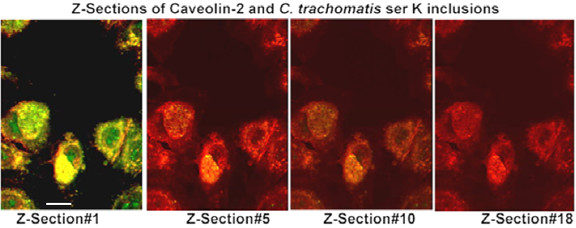
**Optical Z-axis sections of caveolin-2 associated with chlamydial inclusion membranes. **FRT cells were infected with *C. trachomatis *serovar K for 48 h. Cells were fixed with 10% cold methanol and double stained with a guinea pig anti-*Chlamydia *and a mouse anti-caveolin-2 antibody. The secondary antibodies were FITC-conjugated goat anti-mouse and TRITC-conjugated goat anti-guinea pig antibody. Slides were examined using a laser confocal microscope and optical Z-axis sections were taken at 0.5 μm depth and images merged using the Confocal Assistant™ version 4.02 Image Processing Software. Original magnification: 600X; the scale bar is 25 μm in length.

We asked whether caveolin-2 might be associated with the inclusions of each of the ten chlamydial strains and species that we had previously assessed for raft-mediated entry and for inclusion-associated caveolin-1 [19]. We began by looking at infections in caveolin-1 positive HeLa cells. Inclusions of *C. trachomatis *serovars E, F, and K, *C. pneumoniae *(A39), and *C. caviae *(GPIC), previously were seen to enter HeLa cells via lipid rafts, and their inclusions were marked by caveolin-1 in those cells (ibid). Immunostaining with caveolin-2 specific monoclonal antibodies demonstrated that in HeLa cells, inclusions of each of these strains also were marked by caveolin-2 (Figure 1, Table 1). As in the case of caveolin-1 [19], optical sections through the Z-axis clearly indicated the caveolin-2 as packets associated with the vacuolar membrane (Figure 2). The disappearance of the caveolin-2 staining as the depth of the optical sections increased, with no change in the anti-*Chlamydia *staining, confirms that the caveolin-2 proteins are associated with the vacuolar membranes of these inclusions and are not associated with the individual EBs and RBs inside of the chlamydial vacuole. This profile is similar to that seen when Z-sections were taken of the caveolin-1 staining.

Importantly, immunostaining for caveolin-2 appears nonrandom on the inclusion membrane (Figure 2, Z section #1). Indeed, the series of merged optical sections shows that the caveolin-2 staining appears in apposition to those pathogen cells that are at the inclusion membrane (Figure 2, see Discussion).

In contrast to the above strains, *C. trachomatis *serovars A, B, and C, do not enter via lipid rafts, and their inclusions do not acquire caveolin-1 in HeLa cells [19]. Nevertheless, in HeLa cells inclusions of each of these strains also acquired caveolin-2 (Figure 1, Table 1). In our earlier study, entry of strains LGV (L2) and MoPn was seen to be independent of lipid rafts, and their inclusions were not marked by caveolin-1. In the present study, their inclusions did not display caveolin-2 and therefore they did not accumulate this host protein during their development (Figure 1, Table 1).

### Caveolin-2 is present on chlamydial inclusions in caveolin-1 negative FRT cells

**Figure 3:**
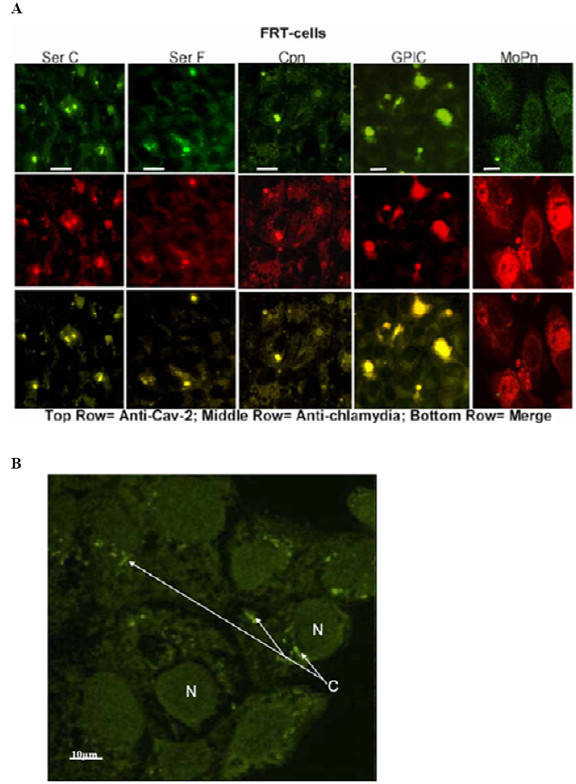
**Acquisition of caveolin-2 by inclusions in caveolin-1 negative FRT cells. (A) **FRT cells were infected with chlamydial organisms for 48 h. *C. pneumoniae*, *C. psittaci*, and *C. trachomatis *serovars A, B, E, K (not shown), as well as serovars C and F colocalized with caveolin-2. MoPn and LGV (not shown), did not colocalize with caveolin-2 in these cells. Note that only two species, the mouse pneumonitis strain (MoPn) and GPIC produce large inclusions in these cells even after 48 h infection (scale bar is 25 μm). The small inclusions nonetheless release viable progeny into the culture supernatant which could be used to infect new cells. (B) Uninfected FRT-cells stained with anti-caveolin-2 antibody to demonstrate the localization of caveolin-2 protein in the cell. Note the caveolin-2 staining (C) close to the nucleus of the cell and that there is no caveolin in the cell membrane. (N), the nucleus of the cells and the scale bar represents 10 μm. Original magnification: 600X

Others have demonstrated that caveolin-2 is not transported out from the Golgi in caveolin-1 negative FRT cells, but that expression of a transfected caveolin-1 gene in those cells restores caveolin-2 transport [25,26]. These experimental findings imply that caveolin-2 transport is dependent on its association with caveolin-1. Thus, it was somewhat surprising that during development in HeLa cells, inclusions of *C. trachomatis *serovars A, B, and C acquired caveolin-2 without acquiring caveolin-1. To confirm that chlamydial inclusions indeed could acquire caveolin-2 independently of caveolin-1, we further examined infections using caveolin-1 negative FRT cells. Normally these cells do not express caveolin-1 or form caveolae, but do so when transfected with caveolin-1 cDNA [27-29]. Inclusions of *C. trachomatis *serovars A, B, C, E, F, and K, as well as *C. pneumoniae*, and *C. caviae *each were marked by caveolin-2 in the caveolin-1 negative FRT cells (Figure 3). This clearly confirms that trafficking of caveolin-2 to chlamydial inclusions does occur independently of caveolin-1. Note that earlier we demonstrated that among those we tested, MoPn and GPIC are the only strains that develop large inclusions in FRT cells, and confirmed that these cells indeed do not express caveolin-1 [19].

**Figure 4:**
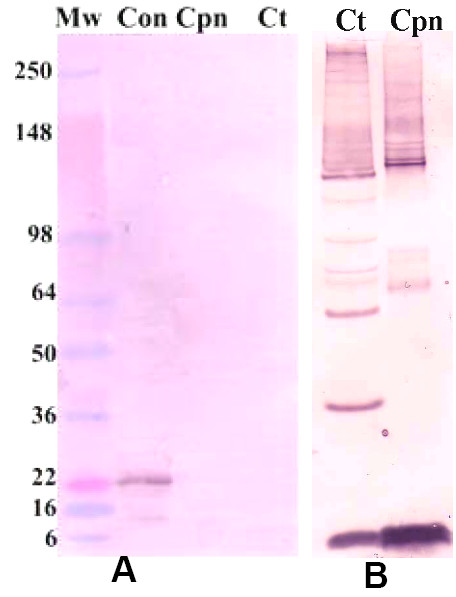
**Anti-caveolin-2 antibodies do not cross-react with chlamydial EBs**. *C. trachomatis *serovar K and *C. pneumoniae *AR39 EBs were purified by renografin gradient and separated on a 10–12% NuPAGE gel. The proteins were transferred to a PVDF membrane, (A) stained with a mouse monoclonal anti-caveolin-2 antibody and the reacting complex detected with an AP-conjugated goat-anti-mouse secondary antibody. Mw is the molecular weight marker, Con is an endothelial cell lysates for caveolin-2 control, Cpn is *C. pneumoniae *EBs and Ct represents *C. trachomatis *EBs. Note that there is staining in the control lane at 22 kDa (caveolin), while there is no staining in the chlamydial organism lanes. (B) control blot using the same quantity and preparation of *C. trachomatis *serovar K and Cpn EBs as above. The blot was stained with a rabbit anti-*Chlamydia *polyclonal antibody.

To confirm the specificity of the caveolin-2 antibody for the caveolin-2 protein in these cells, and to rule out the possibility of cross-reactivity of this antibody with the chlamydial EBs, we performed western blot on renografin gradient purified EBs from *C. pneumoniae *and *C. trachomatis *and stained the blot with anti-caveolin-2 antibodies (figure 4A). As seen in the representative blot, the caveolin-2 antibody does not cross-react with purified EBs. This is also true for anti-caveolin-1 antibodies (data not shown). The presence of chlamydial EBs on the blot was assessed by loading the same amount of EBs onto the second half of the gel in figure 4A and staining with a rabbit anti-*Chlamydia *serum (figure 4B). Note that there is adequate material on this stained blot, confirming that if the caveolin antibody was cross-reactive with EB material one would be able to see such a reaction.

### Acquisition of caveolin-2 by inclusions requires pathogen gene expression

**Figure 5:**
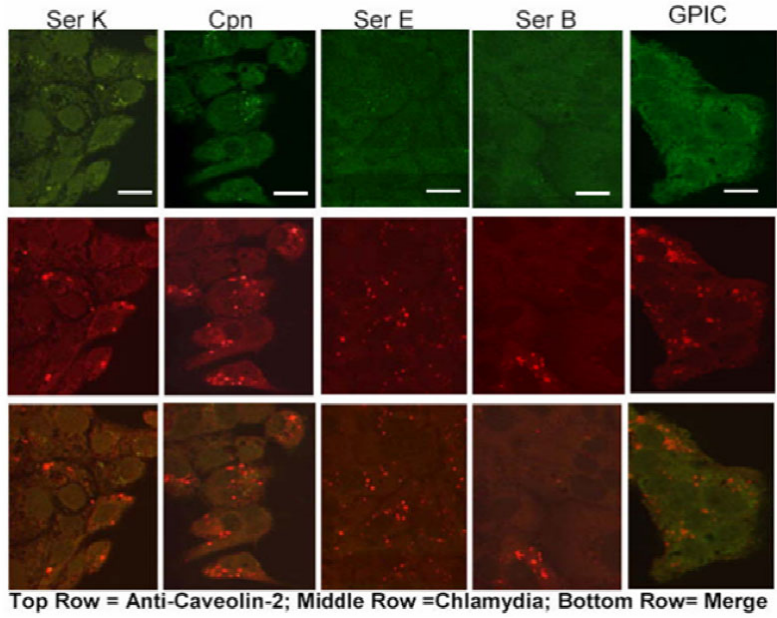
**Ultraviolet-treated EBs do not colocalize with caveolin-2 in caveolin-1 negative FRT cells. **Chlamydial EBs were treated with short wavelength UV for 1 h and then used to infect FRT HeLa cells for 36 h. The cells were fixed and stained with a mouse anti-caveolin-2 and a guinea pig anti-*Chlamydia *antibody. FITC-conjugated goat anti-mouse as well as TRITC-conjugated goat anti-guinea pig secondary antibody was used to visualize the stained EBs. The representative confocal micrographs above show that UV-treated EBs did not colocalize with caveolin-2 in FRT cells. All of the *Chlamydiaceae *not shown had a similar morphology. Scale bar represents 15 μm Original magnification: 600X

**Figure 6:**
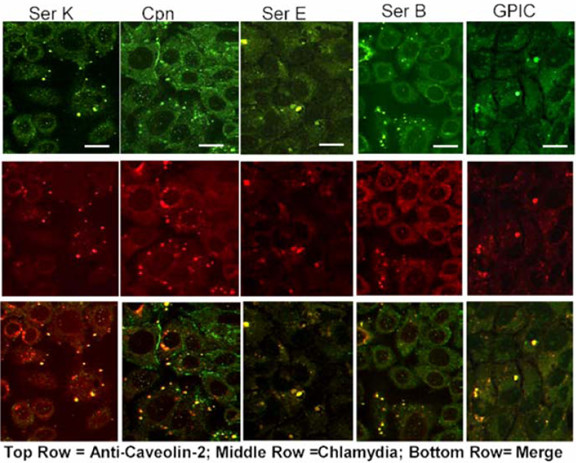
**Ultraviolet-treated EBs colocalize with caveolin-2 in HeLa cells. **Chlamydial EBs were treated with UV light for 1 h and then used to infect HeLa 229 HeLa cells for 36 h. The cells were fixed and stained with a mouse anti-caveolin-2 and a guinea pig anti-*Chlamydia *antibody. A FITC-conjugated goat anti-mouse antibody and a TRITC-conjugated goat anti-guinea pig secondary antibody were used to visualize the stained EBs. Unlike the case in FRT cells, UV-treated EBs colocalized with caveolin-2 in HeLa cells, which express both Caveolin-1 and Caveolin-2. The above confocal micrographs are representative of the morphology of all *Chlamydiaceae *that colocalize with caveolin-2 (*C. pneumoniae*, GPIC, and *C. trachomatis *serovars A, B, C, E, F and K). Original magnification: 600 X. Each scale bar represents 15 μm.

The apparent inability of caveolin-2 to traffic from the Golgi independently of caveolin-1 in uninfected cells [25,26] implies that in a manner yet unknown, the chlamydial inclusions might produce a factor that activates an additional sorting and transport process for caveolin-2. To test this hypothesis, we asked whether UV-inactivated EBs, the infectious form of the pathogen, might sequester caveolin-2 on their vacuoles in caveolin-1 negative FRT cells. Pathogen-containing vacuoles of each of the strains indeed were unable to acquire caveolin-2 when the EBs had been inactivated by UV irradiation prior to infection. This result therefore supports the premise that vacuole/inclusion acquisition of caveolin-2 is dependent on pathogen gene expression (Figure 5). In caveolin-1 positive HeLa cells, a minimal amount of caveolin-2 protein was observed to co-localize to vacuoles with UV-treated EBs (Figure 6, see Discussion).

## Discussion

Experimental results presented here demonstrate that for a number of chlamydial strains and species, the intracellular inclusions indeed acquire caveolin-2 during the developmental cycle. These strains include *C. trachomatis *serovars A, B, C, E, F, and K, *C. pneumoniae *(A39), and GPIC (Table 1). *C. trachomatis *serovars E, F, and K, and the *C. pneumoniae *and *C. caviae *species of *Chlamydophila *previously were shown to enter host cells via lipid rafts. Their vacuoles/inclusions were demonstrated to acquire host cell caveolin-1 at entry and to accumulate it during later stages of infection [18,19].

Consequently, in host cells that express both caveolin proteins, inclusions of these strains display caveolin-2, as well as caveolin-1. However, in this same cell type, inclusions of serovars A, B, and C, are marked only by caveolin-2, a result implying that for chlamydial inclusions this acquisition can occur independently of caveolin-1. This implication is somewhat surprising since there is experimental evidence demonstrating that caveolin-2 usually can not exit from the Golgi and traffic to the plasma membrane in the absence of caveolin-1[25,26]. However, if recombinant caveolin-1 DNA is expressed by caveolin-1 negative cells, then caveolin-2 can transit from the Golgi and be delivered to the plasma membrane, as a hetero-oligomeric complex with caveolin-1[25,26,30,31].

To confirm that transport of caveolin-2 to chlamydial inclusions indeed might occur independently of caveolin-1, we asked whether chlamydial inclusions might similarly acquire caveolin-2 in FRT cells, a cell line that does not express caveolin-1 [27-29]. In our earlier report [19], we confirmed that FRT cells in fact do not express caveolin-1. Also, we demonstrated that whereas for some chlamydial strains, entry into those cells was dependent on intact lipid rafts (Table 1), in no case was entry dependent on caveolin-1 and caveolae. We show here that in the caveolin-1 negative FRT cells, inclusions of each of the above chlamydial strains in fact acquired caveolin-2. Moreover, since FRT cells do not contain caveolin-2 at the plasma membrane, pathogen-associated caveolin-2 could not have been acquired at entry. Rather, it had to derive from an intracellular source.

Chlamydial inclusions thus acquired caveolin-2 despite the fact that in the absence of caveolin-1 this protein usually does not traffic from the Golgi. We therefore suggested that the pathogen might influence the host cell and actually induce a transport and sorting pathway that normally may not actively function in the uninfected cell. Consistent with this suggestion is the fact that for all strains and species tested in FRT cells, if the pathogens were inactivated by prior EB treatment with UV irradiation, none of the EB containing vesicles acquired caveolin-2. In contrast, in HeLa cells, there were low levels of caveolin-2 associated with vesicles containing UV-treated EBs. The source of these low levels of caveolin-2 on vesicles/inclusions in HeLa cells is not yet clear. Perhaps the EBs are acquiring minimal amounts of caveolin-2 from the membranes of these cells upon entry. This suggestion is supported by the fact that the vesicles of these UV irradiated EBs contain both caveolin-1 and -2 (data not shown); suggesting that the proteins might be co-localizing in hetero-oligomeric complexes.

It is well known that *Chlamydia*-containing vacuoles and later, the developing inclusions, are able to intercept and fuse with exocytic vesicles of the biosynthetic pathway that originate from the Golgi [6,7]. These Golgi-derived vesicles provide the inclusions with sphingolipids and perhaps other key metabolites [7,32]. In addition, the ability of at least some chlamydial strains to intercept these Golgi-derived vesicles is dependent on pathogen viability and therefore likely on gene expression. Considering these points, and the fact that caveolin-1/2 hetero-oligomers traffic to the plasma membrane from the Golgi [25,26,30,31], it is reasonable to suggest that the previously described pathogen induced interception of Golgi-derived vesicles might account for the acquisition of caveolin-2 by inclusions, as seen in the current study. However, although studies by others indicate that all chlamydial strains appear to intercept vesicles of the biosynthetic pathway, not all strains acquire caveolin-2 and this fact provides a strong counter argument. Thus for example, in the current study LGV (L2) inclusions did not acquire caveolin-2 (Table 1), although they have been reported to intercept Golgi-derived vesicles of the biosynthetic pathway [7]. Furthermore, in the current study, acquisition of caveolin-2 by inclusions of some strains was independent of caveolin-1, while among other strains, inclusions acquired both caveolin proteins. Thus there may be more than one pathway by which inclusions acquire caveolin-2. Importantly, acquisition of caveolin-2 by inclusions of any particular strain did not correlate with entry by a raft-mediated pathway. In contrast, acquisition of caveolin-1 at early as well as late stages in pathogen development did correlate with a route of entry involving lipid raft microdomains. Together, these facts are consistent with the conclusion that acquisition of caveoloin-1 and -2 can represent independent and distinct processes. As expected, similar incubations with anti-caveolin-3 demonstrated this component was not present in any of these cells whether infected with *Chlamydiae *or not.

To date, no chlamydial protein has been identified that might be secreted into the host cell cytosol to influence host intracellular trafficking. However, several chlamydial proteins, termed Incs, have been identified in the inclusion membrane [33-35]. Several of these Inc proteins have cytoplasmic domains, making them potential mediators for interactions with the host that might influence trafficking [36]. One of these proteins, IncG, interacts with host protein, 14-3-3β [37]. This particular interaction can not underlie the trafficking of caveolin-2 to the inclusions since GPIC (*C. caviae*) and *C. pneumoniae *(AR39) do not express IncG [37], although, as shown here, they do acquire caveolin-2. Furthermore, the reverse also is true. Thus, as demonstrated above, mouse pneumonitis strain, MoPn and LGV (L2) do not acquire caveolin-2, but they do express IncG [37]. There are, however, other Inc proteins and they may underlie the phenomena we have presented here.

Despite their presence on chlamydial inclusions, it remains unclear what role, if any, caveolin proteins may play in the developmental cycle of *Chlamydia*. As noted above, all ten of the chlamydial strains and species we examined were able to enter caveolin-1 negative FRT cells [19]. Since these caveolin-1 negative cells do not express caveolin-2 at the plasma membrane [25,26], it would appear that for any of these strains neither of the caveolin proteins is necessary for entry per se. Although following internalization all ten strains and species remained viable in the FRT cells [19], the only strains able to generate large inclusions in the FRT cells were GPIC which acquires both caveolin-1 and -2 in caveolin-positive cells, and MoPn which acquires neither caveolin protein. Despite these latter two instances, our findings remain consistent with the possibility that caveolin-1 may yet play a post entry role in the development of at least some strains. The fact that successful MoPn development does occur in inclusions that acquire neither caveolin-1 nor -2 [19] (and current study) could mean that neither caveolin-1 nor -2 has a necessary role in the development of MoPn. The ability of GPIC to develop mature inclusions in FRT cells where it acquires only caveolin-2, despite acquiring both caveolin proteins in caveolin-1 positive cells, may imply that for GPIC caveolin-2 rather than caveolin-1 is a key for development. Likewise, inclusions of *C. trachomatis *serovars A, B, and C, whether developing in HeLa or FRT cells, display only caveolin-2, suggesting a potentially broader chlamydial requirement for the caveolin-2 protein during development.

The series of merged optical sections demonstrating the association of caveolin-2 with the inclusions (Figure 2) implies that caveolin-2 is in apposition to pathogen cells that are located at the inclusion membrane. Nevertheless, the caveolin-2 is not in direct contact with the pathogens. Caveolin is not a transmembrane protein, a fact that can be deduced from the experimental finding that cell-surface biotinylation does not label caveolin proteins [38]. Thus, these membrane proteins are not accessible from the extracellular milieu, and this originally extracellular membrane surface, is topologically equivalent to the lumen of the chlamydial inclusion. Hence, whereas our findings imply a specific association between the pathogens and caveolin-2, this association appears to be indirect. The paucity of information concerning caveolin-2 does not enable us to suggest an identity for the linking molecule. Moreover, how this indirect association between caveolin-2 and the pathogen might relate to the protein's function in the chlamydial developmental cycle, or in its acquisition by the pathogen, is also not yet clear.

## Conclusions

Among the *Chlamydiae*, depending on the serovar, or the species, one or both of the caveolin proteins may play an important role in the developmental cycles. Further studies of the interaction of the *Chlamydiae *with these enigmatic host cell proteins may well help to clarify caveolin functions in the host cell. Likewise, such studies may indicate factors influencing caveolin expression and trafficking from the Golgi, and elucidate their significance to the developmental cycle of these pathogens. The function of caveolin-2, either in the uninfected cell or in the chlamydial developmental cycle, remains to be elucidated. Nevertheless, this second caveolin protein can now be added to the small number of host proteins that are reported as associated with the inclusions of this obligate intracellular pathogen.

## Competing interests

None Declared.

## Authors' contributions

ES and LN conceived of the study and participated in its design and coordination. LN was instrumental in the design of the study and prepared the initial draft of the manuscript. WW carried out all the experimental work, took photographs, did statistical analyses where necessary, and participated in the design and coordination of the project. All authors contributed to the preparation of the final manuscript.

## Pre-publication history

The pre-publication history for this paper can be accessed here:



## Figures and Tables

**Table 1:** Comparative colocalization of the *Chlamydiaceae *with caveolin-1 and -2 Note that caveolin-2 colocalizes with all the inclusions that caveolin-1 colocalized with. In addition, caveolin-2 is seen associated with the inclusions of serovars, A, B and C, all ocular serovars, which did not colocalize with caveolin-1

**Organisms**	**Caveolin-1**	**Caveolin-2**	**Raft-mediated Entry**
***C. pneumoniae ***(AR 39)	+	+	Yes
***C. caviae ***(GPIC)	+	+	Yes
***C. trachomatis ***(MoPn)	-	-	No
***C. trachomatis ***(ser A)	-	+	No
***C. trachomatis ***(36 B)	-	+	No
***C. trachomatis ***(ser C)	-	+	No
***C. trachomatis ***(ser E)	+	+	Yes
***C. trachomatis ***(ser F)	+	+	Yes
***C. trachomatis ***(ser K)	+	+	Yes
***LGV (L2)***	-	-	NO

(+) colocalization; (-) no colocalization
